# Intravenous Lobular Capillary Haemangioma (Pyogenic Granuloma) of the Superior Vena Cava: Case Report and Literature Review

**DOI:** 10.1016/j.ejvsvf.2020.12.021

**Published:** 2020-12-24

**Authors:** Elisabeth Blaya, Vincenzo Vento, Salomé Kuntz, Laurence Bruyns, Mickael Ohana, Noelle Weingertner, Anne Lejay, Nabil Chakfé

**Affiliations:** a Department of Vascular Surgery and Kidney Transplantation, University of Strasbourg, France; b GEPROVAS, Groupe Européen de Recherche sur les Prothèses appliquées à la chirurgie vasculaire, Strasbourg, France; c Department of Radiology, University of Strasbourg, France; d Department of Pathology, University of Strasbourg, France

## Abstract

**Introduction:**

Intravascular lobular capillary haemangioma is a rare benign intravascular tumour, especially in large vessels. This is the report of a case and associated literature review.

**Report and literature review:**

This is the report of the first case of an intravenous lobular capillary haemangioma (ILCH) of the superior vena cava (SVC). A 30 year old female presented with a collateral thoraco-abdominal venous circulation. Chest computed tomography angiography, thoracic magnetic resonance imaging, and positron emission tomography revealed an intraluminal SVC tumour extending from the left brachiocephalic venous trunk to the distal third of the SVC. No pre-operative biopsy was indicated. An *en bloc* tumour excision was performed, followed by reconstruction of the SVC with an L shaped, ringed polytetrafluoroethylene (PTFE) prosthesis. Histopathology revealed the presence of an ILCH with free margins. A review of the literature identified 64 cases of ILCH to date, all of which underwent total resection. When reported, no recurrences were found during follow up.

**Discussion:**

In this case, the ePTFE reconstruction of the SVC must be checked regularly for any adverse events. Although ILCH is a benign tumour with no risk of recurrence, regular surveillance is advised.

## Highlights

• Report of the first case of intraluminal capillary haemangioma involving the superior vena cava.
• Literature review of intraluminal capillary haemangioma.
• Successful *en bloc* tumour excision followed by an L shaped prosthetic repair.

## Introduction

Primary vascular tumours of the vena cava are rare and occur mainly in the inferior vena cava (IVC). The ISSVA Classification describes two main types of malignant vascular tumours: angiosarcoma, (mainly leiomyosarcoma, generally in the IVC, none in superior vena cava [SVC]) and epithelioid haemangio-endothelioma. Lobular capillary haemangioma (LCH), also known as pyogenic granuloma, is one of the many benign vascular tumours. It occurs mainly in the skin and subcutaneous tissue of the upper limb, neck, and head. Intravascular lobular capillary haemangioma (ILCH) is a subtype of LCH and only few cases have been reported in the literature.^1^^,^^2^

This is the first case of intravenous lobular capillary haemangioma in the superior vena cava (SVC) reported in the literature. A technique is described for reconstruction of the SVC using an inverse L shaped synthetic graft, after an *en bloc* resection of the tumour.

## Case report

A 30 year old female, without significant medical history, presented with recent aggravation of a thoraco-abdominal venous circulation developed during her pregnancy, five years earlier. She had no other complaint or symptom. Clinical abdominal and cardiovascular examinations did not reveal any specific clinical signs of portal hypertension or SVC syndrome.

**Figure 1:**
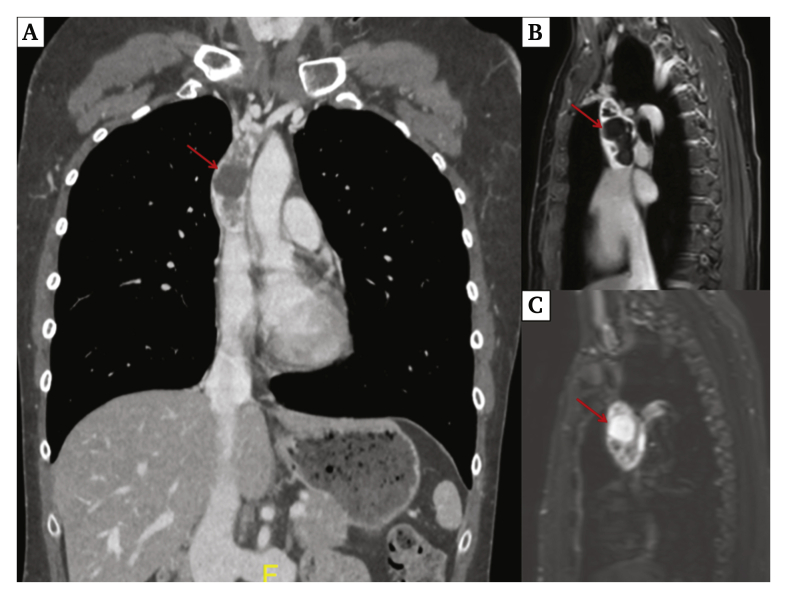
(A) Thoracic computed tomography angiography, coronal reconstruction showing the tumour (red arrow) filling the superior vena cava lumen. (B) Thoracic magnetic resonance imaging T2 weighted, and (C) T1 weighted with gadolinium injection.

Duplex ultrasound (DUS) showed dilatation of collateral veins, while the IVC was patent. Chest computed tomography angiography (CTA) (Revolution Evo G.E. Medical, Chicago, IL, USA) demonstrated an intraluminal heterogeneous mass in the SVC, extending from the left brachiocephalic venous trunk to the distal third of the SVC (Fig. 1A). No other mass or adenopathy was present. Thoracic magnetic resonance imaging (Ingenia Philips 3T, Eindhoven, the Netherlands) confirmed the intraluminal SVC location of the tumour, measuring 80 × 33 mm, with a cystic portion demonstrating heterogeneous T1 weighted and T2 weighted signal (Fig. 1B and C). The azygos vein had not been invaded. Positron emission tomography (Vision Siemens, Munich, Germany) demonstrated moderate fluorodeoxyglucose enhancement (SUV max: 4.8). Transthoracic sonography confirmed no extension into the right atrium. The oncology multidisciplinary staff reviewed the preliminary findings and suspected a primary SVC tumour or SVC thrombosis. A direct biopsy was too risky in terms of spread if malignant, and an endovascular biopsy would not have been sufficient. It was decided to perform surgical resection without radiotherapy or chemotherapy.

**Figure 2:**
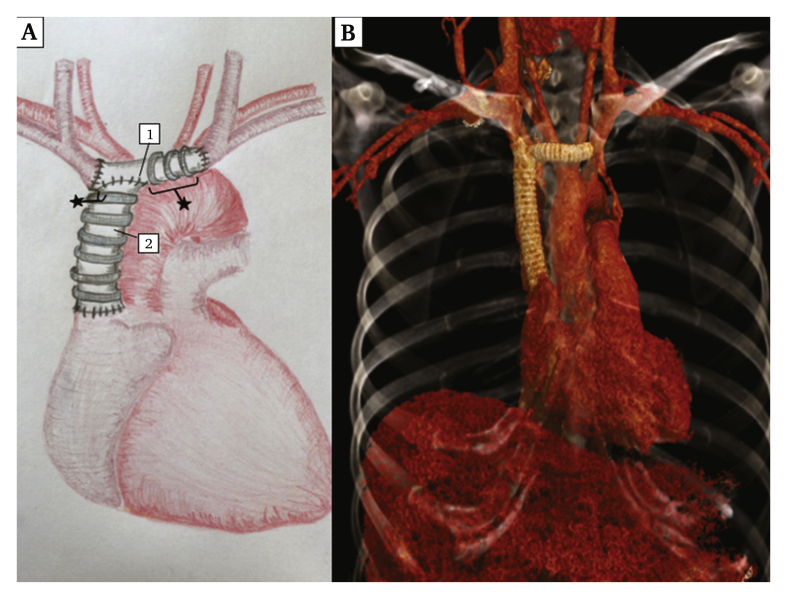
(A) Post-operative schematic representation of the superior vena cava reconstruction with 1) 8 mm polytetrafluoroethylene graft and 2) 16 mm polytetrafluoroethylene graft. (B) Post-operative thoracic computed tomography angiography 3D reconstruction.

Surgery consisted of an *en bloc* resection of the tumour through a median sternotomy. The SVC was resected from its junction with the right atrium without myocardial breach and taking both left and right brachiocephalic veins. The venous reconstruction consisted of a 16 mm diameter, ringed, expanded polytetrafluoroethylene (ePTFE) prosthesis (Gore-Tex, W.L. Gore & Associates, Flagstaff, AZ, USA) sutured distally to the right atrium junction and proximally, perpendicularly on an 8 mm diameter ringed ePTFE prosthesis, connecting both right and left venous confluences (Fig. 2A). There was no need for cardiopulmonary bypass during the intervention. A left brachiobasilic fistula was created to increase blood flow and avoid thrombosis of the prosthesis. The post-operative course was complicated by a pericardial effusion with signs of right heart failure, without any complications of the venous reconstruction. A pericardial drain was placed as an emergency. The patient was discharged on day 26 with anticoagulant therapy (warfarin 7.5 mg once a day), as recommended in venous bypass. Six month DUS and CTA follow up showed graft patency. Fistula closure and anticoagulation interruption were conducted seven months post-operatively.

**Figure 3:**
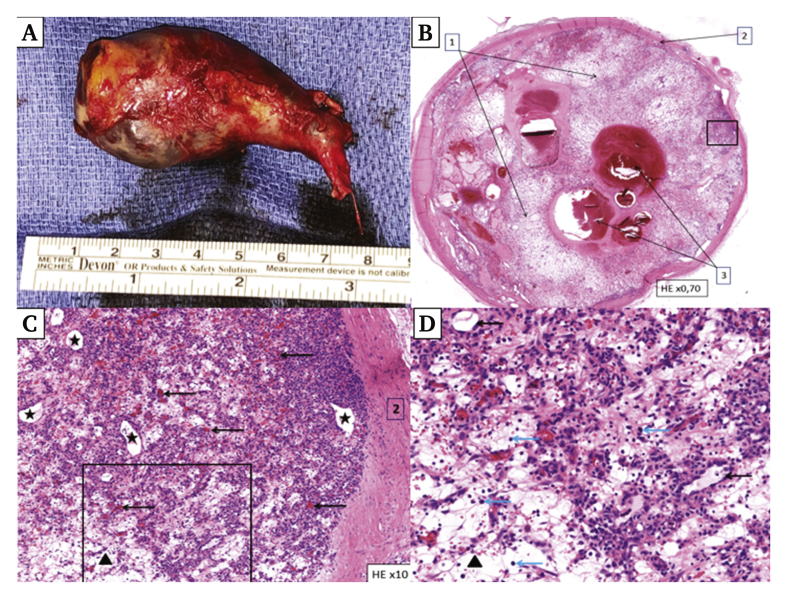
(A) Macroscopic view of the superior vena cava specimen: the tumour was strictly intraluminal. (B) Histology of a transverse cross section of the tumour demonstrated an intravenous (venous wall [1]) mass obstructing the lumen (2) with lobular architecture and thrombotic re-organisation (3) (haematoxylin and eosin stain [H&E] ×0.70). (C) Close up picture of the mass showed numerous small capillaries (arrows) and larger vessels (black stars) within fibro-oedematous stroma (black triangle) (H&E ×10). (D) At higher magnification, capillaries showed unstratified endothelium without atypia (black arrow), accompanied by inflammatory cells (blue arrow) (H&E ×20).

Pathological examination showed a tumour measuring 55 × 34 × 25 mm, filling the SVC lumen (Fig. 3A and B). Microscopic analysis identified a vascular lesion with lobular architecture, consisting of multiple capillaries lining unstratified endothelium without atypia separated by a fibro-oedematous stroma (Fig. 3C and D). Immunohistochemical analysis showed vascular markers, CD31 and ERG, characterising the endothelium. No sign of malignancy was found and the Ki67 proliferation index was low. After confirmation with the *Réseau de Référence en Pathologie des Sarcomes des Tissus Mous* (National French Reference Network of Soft Tissues Sarcomas), the final diagnosis was an ILCH in the SVC, also known as pyogenic granuloma.

## Literature review

A systematic search was performed of the Medline database from 1979 to 2020 by a combined search strategy of MeSH terms (intravenous lobular capillary haemangioma, pyogenic granuloma). All titles and abstracts collected from the search strategy were screened for relevance. The first 20 related items of all relevant articles were scanned for other potentially relevant studies. Full texts of all relevant articles were obtained and reviewed for relevance. The reference lists of each article were scanned for other potentially relevant studies. The systematic search identified 43 full text English studies, one full text German study, and one full text Korean study, including 39 single case reports, two case series, and four reviews, corresponding to a total of 64 cases.

Ten cases (15.6%) were symptomatic, with symptoms such as abdominal pain, limb oedema, or pleural effusion, six (9.4%) were incidental findings after routine examinations, and 48 (75%) presented with nodules, pain free or not. The male/female ratio was 10:9 and the median age was 44 years old (interquartile range [IQR] 35, 51).

The treatment was not reported in two cases (3.1%). Total resection was the chosen treatment for 61 cases (98.3%), only one case (1.7%) had a biopsy and regressed spontaneously. Follow up was reported in 22 cases and all had no recurrence, from two months (see reference ^30^ in Supplementary material) to eight years of follow up.^4^

## Discussion

Primary SVC tumours are rare. Three cases of epithelioid haemangio-endothelioma of the SVC and 12 leiomyosarcomas have been reported in the literature.^2^^,^^3^ No cases of SVC ILCH were reported.

LCH was described for the first time in 1979.^4^ ILCH is a rare type of LCH as, according to the present literature review, only 64 cases have been reported to date (Table 1). This benign vascular tumour is derived from endothelial cells, characterised by an anarchic capillary proliferation in a fibromyxoid oedematous stroma, with endothelial markers such as CD31 and/or CD34, or smooth muscle alpha-actin. Differential diagnoses of ILCH are other intraluminal lesions such as venous thrombosis, papillary endothelial hyperplasia, intravenous atypical vascular proliferation, histiocytoid haemangioma, and angiosarcoma.^1^

**Table 1:** Review of literature regarding intraluminal capillary haemangioma.∗

Authors, date	Sex/age	Anatomical location	Clinical data	Therapy	Outcome data
Cooper, 1979^4^	18 cases NR	Neck×6 Arm×2 Forearm×6 Wrist×2 Finger×2	Not specified	Surgical resection	No recurrence Average of follow up 8.2 y
Ulbright, 1980^9^	M/12	External jugular vein	Neck nodule	Surgical resection	No recurrence at 1 y
Anderson, 1985^10^	F/62	Palmar vein	Palmar nodule	Surgical resection	No recurrence at 21 mo
Truong, 1985^11^	M/44	Branch of angular vein	Lachrymal sac nodule	Surgical resection	No recurrence at 7 y 11 mo
	M/68	Not precise	Inner canthus nodule	Surgical resection	No recurrence at 4 y 7 mo
DiFazio, 1989^12^	F/37	Palmar vein	Palmar nodule	Surgical resection	No recurrence at 16 mo
Saad, 1993^13^	M/35	Left temple	Nodule	Surgical resection	NR
Margo, 1994^14^	M/27	Temporal artery	Temporal nodule	NR	NR
Pesce, 1996^15^	M/20	Lip vein	Nodule	Surgical resection	NR
Danz, 1997^16^	M/79	Portal vein	Asymptomatic – autopsy finding	NR	NR
Hull, 1999^17^	M/73	Renal vein	Asymptomatic – investigation for benign prostate hyperplasia	Total nephrectomy	NR
Domanski, 1999^18^	F/15	Neck vein	Neck nodule	Surgical resection	NR
Sarteschi, 1999^19^	F/56	External jugular vein	Neck nodule	Surgical resection	No recurrence at 2 y
Song, 2001^20^	M/43	External jugular vein	Neck nodule	Surgical resection	NR
Qian, 2001^21^	F/26	Forearm vein	Forearm nodule	Surgical resection	NR
Hayashi, 2001^22^	F/35	Thenar vein	Thenar nodule	Surgical resection	NR
Panchagnula, 2001^23^	F/12	Neck vein	Neck nodule	Surgical resection	No recurrence at 1 y 6 mo
Kocer, 2003^24^	F/58	Palm vein	Palm nodule	Surgical resection	No recurrence at 5 mo
Ghersin, 2004^25^	M/21	Basilic vein	Elbow nodule	Surgical resection	NR
Hung, 2004^26^	F/44	Palmar vein	Ulcerative nodule of the palm	Surgical resection	No recurrence at 6 mo
Ghekiere, 2005^27^	M/50	Cephalic vein	Forearm nodule	Surgical resection	NR
Madison, 2006^28^	M/20	Superficial palmar branch of radial artery	Thenar nodule	Surgical resection	NR
Jung, 2008^29^	M/51	Cephalic vein	Forearm nodule	Surgical resection	NR
Pradhan, 2008^30^	F/75	Right internal iliac vein	Abdominal pain and diarrhoea	Surgical resection	No recurrence at 2 mo
Vijayan, 2008^31^	M/16	Superficial vein	Finger nodule	Surgical resection	No recurrence at 18 mo
Kamishima, 2009^32^	F/56	Non-specified vein	Finger nodule	Surgical resection	NR
	M/66	Non-specified vein	Finger nodule	Surgical resection	NR
Winn, 2009^33^	F/47	Angular vein	Medial canthus nodule	Surgical resection	No recurrence
Maher, 2010^34^	F/41	External jugular vein	Supraclavicular fossa nodule	Surgical resection	NR
Joethy, 2011^35^	M/32	Non-specified	Finger nodule	Surgical resection	No recurrence at 7 mo
Johnson, 2011^36^	F/12	Finger vein	Finger swelling	Surgical resection	No recurrence at 1 ½ mo
Wu, 2011^37^	F/38	Internal jugular vein	Neck nodule	Surgical resection	No recurrence at 1 y
Trombetta, 2011^38^	M/59	Right renal vein	Incidental finding	Total nephrectomy	No recurrence at 5 mo
Takeuchi, 2012^39^	F/36	Left renal vein	Abdominal pain	Total nephrectomy	No recurrence at 4 mo
Turtay, 2012^40^	M/34	Arteriovenous fistula	Pain and swelling of ankle	Surgical resection	NR
Ahn, 2013^41^	F/39	Cephalic vein	Forearm nodule	Surgical resection	NR
Taguchi, 2013^42^	M/53	Forearm vein	Painful forearm nodule	Surgical resection	NR
Umari, 2013^43^	M/50	Renal vein	Asymptomatic – investigation benign prostate hyperplasia	Total nephrectomy	No recurrence at 9 mo
Risio, 2013^44^	F/55	Adrenal gland vein	Abdominal discomfort, anorexia, and nausea	Right adrenalectomy	Uneventful No follow up mentioned
Cera, 2014^45^	M/51	Internal jugular vein	Incidental finding	Surgical resection	Graft patency at 1 mo
Nguyen, 2014^46^	F/79	Azygos vein	Right pleural effusion	Surgical resection	NR
Matsuzaki, 2016^47^	M/73	Right subclavian vein	Right upper arm oedema	Surgical resection	No recurrence
Gameiro, 2016^48^	M/54	Penile corpus spongiosum	Nodule of penal coronal sulcus	Partial biopsy	Regression within 2 weeks No recurrence at 6 mo
Loftus, 2017^2^	M/51	Subcutaneous vein	Forearm nodule	Surgical resection	No recurrence at 10 mo
Bongiolatti, 2018^49^	F/32	Left subclavian vein	Left arm and face oedema	Surgical resection	No recurrence at 1 y
Reimold, 2019^50^	F/78	Right renal vein	Asymptomatic, routine check up	Total nephrectomy	NR
Woo, 2019^51^	M/31	Jugular vein	Neck mass	Surgical resection	No recurrence at 6 mo

NR = not reported; y = years; F = female; M = male; mo = months.

∗ References 9–51 are listed in Supplementary material.

Despite being a benign tumour, surgical removal of ILCH is the first choice approach to make a histological diagnosis and to prevent tumour related complications, such as SVC occlusion or thrombosis, local compression and pulmonary embolisation. As SVC resection is not a frequent procedure, the replacement strategy remains controversial. The main risk is graft thrombosis, occurring between one and five months after implantation.^5^, ^6^, ^7^ Long term patency rates can vary depending on graft material, length, or shape (ePTFE or a biological graft like bovine pericardium). In 2019, Maurizi et al. reported the results of SVC reconstruction with either bovine pericardial conduit (12 cases) or ePTFE grafts (13 cases). In their experience, follow up showed no statistical difference in terms of graft patency.

In this case, an ePTFE vascular graft was chosen. As shown in Fig. 2, the graft was L shaped. A left brachiobasilic fistula was created to increase blood flow and prevent graft thrombosis.

Despite the excellent long term prognosis of the benign characteristics of the tumour, immediate post-operative anticoagulant treatment and close follow up with DUS of the graft will be mandatory to assess graft patency.^8^

### Conclusion

This case reports successful management of *en bloc* resection of an SVC ILCH associated with SVC prosthetic reconstruction.

## Appendix A Supplementary data

Multimedia compoennt 1
