# Supplementary material for: Intravenous Lobular Capillary Haemangioma (Pyogenic Granuloma) of the Superior Vena Cava: Case Report and Literature Review
Source: EJVES Vasc Forum. 2020 Dec 24;50:32–6. doi: 10.1016/j.ejvsvf.2020.12.021 (PMC8074681; doi:10.1016/j.ejvsvf.2020.12.021)
Supplement: Multimedia compoennt 1 [file mmc1.docx]

**Intravenous lobular capillary hemangioma (pyogenic granuloma) of the superior vena cava – Case report and Literature review**

References of literature review (Additional material)

9. Ulbright TM, Santa Cruz DJ. Intravenous pyogenic granuloma: case report with ultrastructural findings. Cancer 1980; 45: 1646–52.

10. Anderson WJ. Intravenous pyogenic granuloma of the hand. J Hand Surg 1985; 10:728–9.

11. Truong L, Font RL. Intravenous pyogenic granuloma of the ocular adnexa. Report of two cases and review of the literature. Arch Ophthalmol 1985; 103: 1364–7

12. DiFazio F, Mogan J. Intravenous pyogenic granuloma of the hand. J Hand Surg 1989; 14: 310–2.

13. Saad RW, Sau P, Mulvaney MP, James WD. Intravenous pyogenic granuloma. Int J Dermatol 1993; 32: 130–2

14. Margo CE. Intravascular hemangioma of the temporal artery. Arch Ophthalmol 1994; 112: 1024

15. Pesce C, Valente S, Gandolfo AM, Lenti E. Intravascular lobular capillary haemangioma of the lip. Histopathology 1996; 29: 382–4.

16. Danz M, Mentzel T. Intramurales Hämangiom der Vena portae. Pathol 1997; 18: 243–5.

17. Hull GW, Genega EM, Sogani PC. Intravascular capillary hemangioma presenting as a solid renal mass. J Urol 1999; 162: 784–5.

18. Domanski HA. Intravenous pyogenic granuloma mimicking pleomorphic adenoma in a fine needle aspirate. A case report. Acta Cytol 1999; 43: 439–41.

19. Sarteschi LM, Bonanomi G, Mosca F, Ferrari M. External jugular vein hemangioma occurring as a lateral neck mass. J Ultrasound Med 1999; 18: 719–21.

20. Song MG, Kim HJ, Lee ES. Intravenous pyogenic granuloma. Int J Dermatol 2001; 40: 57–9.

21. Qian LH, Hui YZ. Intravenous Pyogenic Granuloma: Immunohistochemical Consideration A Case Report. Vasc Surg 2001; 35: 5.

22. Hayashi H, Shimizu T, Matsumura T, Shimizu H. Intravenous Pyogenic Granuloma of the Hand. Acta Derm Venereol 2001; 81: 313–4

23. Panchagnula R, Kini U. Intravascular Lobular Capillary Hemangioma Arising in an Hemangioma: A much Disputed Entity. Otolaryngol Neck Surg 2001; 125: 574–5.

24. Kocer U, Aksoy HM, Ozer Tiftikcioglu Y, Karaaslan O. Intravenous Pyogenic Granuloma of the Hand. Dermatol Surg 2003; 29: 974–6.

25. Ghersin E, Nitecki S, Brook OR, Ofer A, Kaftori JK, Hoffman A, et al. Intraluminal pyogenic granuloma of the basilic vein: color duplex sonographic manifestations. J Ultrasound Med Off J Am Inst Ultrasound Med 2004; 23: 443–5.

26. Hung CH, Kuo HW, Chiu YK, Huang PH. Intravascular pyogenic granuloma arising in an acquired arteriovenous malformation: report of a case and review of the literature. Dermatol Surg 2004; 30: 1050–3.

27. Ghekiere O, Galant C, Vande Berg B. Intravenous pyogenic granuloma or intravenous lobular capillary hemangioma. Skeletal Radiol 2005; 34: 343–6.

28. Maddison A, Tew K, Orell S. Intravenous lobular capillary haemangioma: Ultrasound and histology findings. Australas Radiol 2006; 50: 186–8.

29. Jung JY, Goo B, Choi YJ, Chung HJ, Chung KY. A case of granuloma pyogenicum presenting as an intravascular mass: evaluation by ultrasonography. J Eur Acad Dermatol Venereol 2008; 22: 515–7.

30. Pradhan S, Bazan H, Salem R, Gusberg RJ. Intravenous lobular capillary hemangioma originating in the iliac veins: A case report. J Vasc Surg 2008; 47: 1346–9.

31. Vijayan S, Vijayan K. Intravenous pyogenic granuloma – a case report. Int J Orthoped Surg 2008; 11: 2

32. Kamishima T, Hasegawa A, Kubota KC, Oizumi N, Iwasaki N, Minami A, et al. Intravenous pyogenic granuloma of the finger. Jpn J Radiol 2009; 27: 328–32.

33. Winn BJ, Herreid PA, Sires BS. Intravenous pyogenic granuloma of the angular vein. Ophthal Plast Reconstr Surg 2009; 25: 341–3.

34. Maher A. Intravenous Lobular Capillary Hemangioma. Ann Vasc Surg 2010; 24: 951.e13-951.e15.

35. Joethy J, Al Jajeh I, Tay SC. Intravenous pyogenic granuloma of the hand - a case report. Hand Surg 2011; 16: 87-9.

36. Johnson NA, Haeney J, Yii NW. Intravenous pyogenic granuloma of the finger. J Hand Surg Eur Vol 2011; 36: 251-2.

37. Wu Z, Cheng W, Wang S, Chen Z. Pyogenic granuloma in the internal jugular vein. Eur Heart J 2011; 32: 772–3.

38. Trombetta C, Bertolotto M, Bussani R. Intravenous pyogenic granuloma originating in the renal vein: letter to the editor. Int J Urol 2011; 18: 869–70.

39. Takeuchi M, Hara S, Itoh T. A case of intravenous lobular capillary hemangioma of the renal vein mimicking renal cell carcinoma: Letter to the Editor. Pathol Int 2012; 62: 441–3.

40. Turtay MG, Samdanci E, Oguzturk H, Colak C, Dogan M. A rare cause of ankle pain: concomitant intravenous lobular capillary haemangioma and arteriovenous fistula. Eur Rev Med Pharmacol Sci 2012; 16: 1120–2.

41. Ahn SE, Park JS, Ryu KN, Jin W, Park SY. Intravenous lobular capillary hemangioma occurring in the cephalic vein of a 39-year-old adult. J Clin Ultrasound 2014; 42: 375–8.

42. Taguchi S, Kakefuda T, Ono S, Matsuda S, Mori A, Narimatsu Y, et al. Intravenous Lobular Capillary Hemangioma Occurring after Needle Insertion during Routine Health Checkup. Ann Vasc Dis 2013; 6: 102–5.

43. Giovanni Liguori PU. An Atypical Site of Vascular Tumor: IVPG of Right Renal Vein. J Vasc Med Surg 2013; 01(04).

44. Risio D, Selvaggi F, Viola P, Lattanzio G, Legnini M, D’Aulerio A, et al. Intravenous pyogenic granuloma of the right adrenal gland: report of a case. Surg Today 2013; 43: 569–73.

45. Cera C, Calvagna C, Sgorlon G, Zamolo F, Pancrazio F, Adovasio R. Internal Jugular Vein Pyogenic Capillary Hemangioma: A Case Report. Ann Vasc Surg 2015; 29: 361.e5-361.e7.

46. Nguyen BD. Imaging of Intravenous Lobular Capillary Hemangioma of Azygos Vein: Clin Nucl Med 2014; 39: e114–6.

47. Matsuzaki K, Imamura Y, Ozawa M, Nakajima T, Ikeda A, Konishi T, et al. Intravenous Lobular Capillary Hemangioma in the Subclavian Vein. Ann Thorac Surg 2016; 102: e427–9.

48. Gameiro A, Cardoso JC, Calonje E, Tellechea O. Intravascular Lobular Capillary Hemangioma in the Corpus Spongiosum. Am J Dermatopathol 2016; 38: e15-17.

49. Bongiolatti S, Massi D, Maio V, Gonfiotti A, Viggiano D, Voltolini L. Venous outlet syndrome caused by capillary hemangioma of the subclavian vein. Asian Cardiovasc Thorac Ann 2018; 26: 224–6.

50. Philipp R, Jonas L, Georgi T, Cem A, Claudia G, Joanne Nyaboe N-D. Intravenous pyogenic granuloma of the renal vein: A rare morphological differential diagnosis to renal cell carcinoma. Urol Case Rep 2019; 26: 100951.

51. Kim SW, Kim SY, Noh SH, Lee SH. A Case of Intravenous Pyogenic Granuloma Originating in the External Jugular Vein. Korean J Otorhinolaryngol-Head Neck Surg 2019; 62: 307–11.
